# Balance between Estrogens and Proinflammatory Cytokines Regulates Chemokine Production Involved in Thymic Germinal Center Formation

**DOI:** 10.1038/s41598-017-08631-5

**Published:** 2017-08-11

**Authors:** Nadine Dragin, Patrice Nancy, José Villegas, Régine Roussin, Rozen Le Panse, Sonia Berrih-Aknin

**Affiliations:** 1 Inovarion, Paris, France; 2 Sorbonne Universités, UPMC Univ Paris 06, Paris, France; 3 INSERM U974, Paris, France; 4 Department of Pathology, New York University, School of Medicine, New York, USA; 5 AIM, institute of myology, Paris, France; 6 Hôpital Marie Lannelongue, Le Plessis-Robinson, France

## Abstract

The early-onset form of Myasthenia Gravis (MG) is prevalent in women and associates with ectopic germinal centers (GCs) development and inflammation in the thymus. we aimed to investigate the contribution of estrogens in the molecular processes involved in thymic GCs formation. We examined expression of genes involved in anti-acetylcholine receptor (AChR) response in MG, MHC class II and α-AChR subunit as well as chemokines involved in GC development (CXCL13, CCL21and CXCL12). In resting conditions, estrogens have strong regulatory effects on thymic epithelial cells (TECs), inducing a decreased protein expression of the above molecules. In knockout mouse models for estrogen receptor or aromatase, we observed that perturbation in estrogen transduction pathway altered MHC Class II, α-AChR, and CXCL13 expression. However, in inflammatory conditions, estrogen effects were partially overwhelmed by pro-inflammatory cytokines. Interestingly, estrogens were able to control production of type I interferon and therefore play dual roles during inflammatory events. In conclusion, we showed that estrogens inhibited expression of α-AChR and HLA-DR in TECs, suggesting that estrogens may alter the tolerization process and favor environment for an autoimmune response. By contrast, under inflammatory conditions, estrogen effects depend upon strength of the partner molecules with which it is confronted to.

## Introduction

Myasthenia gravis (MG) is a heterogeneous neurological autoimmune disease caused by antibodies directed against proteins of the neuromuscular junction. In 85% of patients, antibodies are directed against the acetylcholine receptor (AChR)^1,2^ and associated with thymic abnormalities including follicular hyperplasia and thymoma. Thymic follicular hyperplasia form affects mainly female patients (ratio 4:1) during the fecund period of their life^3^. Of note, thymus removal (thymectomy) completed in an early stage of the disease, is generally an efficient therapy inducing a gradual decrease in anti-AChR antibody titer in the serum and improving symptoms^4,5^.

We and other groups have demonstrated that the biological hallmark of MG thymic hyperplasia is the presence of ectopic germinal centers (GCs)^6^, which provide specific activated and differentiated B cells producing anti-AChR antibodies^7^. These features go along with dysregulated expression of CXCL13, CCL21, and SDF-1/CXCL12^8–10^, chemokines that play a central role in lymphocyte trafficking to lymphoid and non-lymphoid tissues in both physiological^11^ and pathophysiological^12^ conditions.

In autoimmune diseases, the migration and the accumulation of lymphocytes in the target organs are important steps of the pathogenesis. Indeed, high levels of chemokines or cytokines are observed in blood or tissues of patients and correlate with the severity of the disease^8,9,13,14^. Moreover, the ectopic expression of CXCL13 and CCL21 in transgenic mice has been reported to be sufficient to induce lymphoid neogenesis, which leads to the formation of lymph node-like structures^15^. Also, we have recently demonstrated that inflammation is required to reinforce CXCL13 function in recruitment and induction of B cells in tertiary lymphoid organ development^16^.

Interestingly enough, hyperplasic MG thymuses display signs of inflammation with an increased expression of interferon (IFN) type I and type II regulated genes including HLA-DR genes^17^.

Autoimmune MG as compared to most autoimmune diseases (AIDs) are more prevalent in women than men^18^. The reason for this gender incidence was not understood for a long period. Recently, various investigators brought data to light up this phenomenon. Hence, we have demonstrated that the female main sexual hormone, estrogens contribute to the gender bias female susceptibility to AIDs by partially silencing the autoimmune regulator (AIRE)^19^. In addition, estrogens display various known roles in humoral and cellular responses to infection and vaccination in men and women^20^. As well, another group has demonstrated that dihydrotestosterone (DHT), the main testosterone metabolite may have an opposite effect by stimulating in the thymus tolerance mechanisms, again highlighting the role of sexual hormones in AID susceptibility phase. While sex hormones contribute to disease etiology, they also modulate the activity of the immune system and consequently the evolution of AIDs. Therefore, during pregnancy or menstruations, steroid hormones favor a polarization of the immune response towards a Th2 response^21^. For instance in autoimmune MG patients, a worsening of the clinical symptoms during pregnancy or menstruations have been reported, a phenomenon that disappears after thymectomy^22^. Further, we have demonstrated that estrogen receptor subunit α (ER-α) is upregulated in thymocytes of MG patients^23^, suggesting a possible role of sex hormones in thymic pathogenesis and pathology incidence.

Women affected with autoimmune MG are more prone to develop hyperplasic thymuses^3^. Also, and as indicated above, less efficient thymic tolerance process could contribute to the higher female susceptibility to autoimmune MG. Here, we attempt to understand why females display more frequently a hyperplastic thymic in MG, and the involvement of female sexual hormone, estrogens, in these physiopathological processes. To this end, we analyzed the influence of, estrogens on the expression of the chemokines CXCL13, CCL21 and CXCL12 that are highly attractive for B and T cells and are involved in GC formation^24,25^, as well as molecules that participate in the mechanism of central tolerance including HLA class II antigens and AChR subunits.

Our data clearly indicate that estrogens induced a low steady state expression level of most molecules analyzed. However, in an inflammatory environment, comparable to events found in MG thymus, estrogens sustain the activation of interferon-signaling pathways enhancing the process of GC formation.

## Results

### Estrogen effects on HLA-DR and α-AChR expressions in thymus

To understand the molecular differences underlying the female prevalence in MG, we first investigated in normal thymuses from males and females the expression of α-AChR and HLA-DR molecules essential for central tolerance mechanisms, and for the autoantigen presentation in MG. We, then evaluated the effects of estrogens on the expression of these molecules at the mRNA and protein levels.

**Figure 1:**
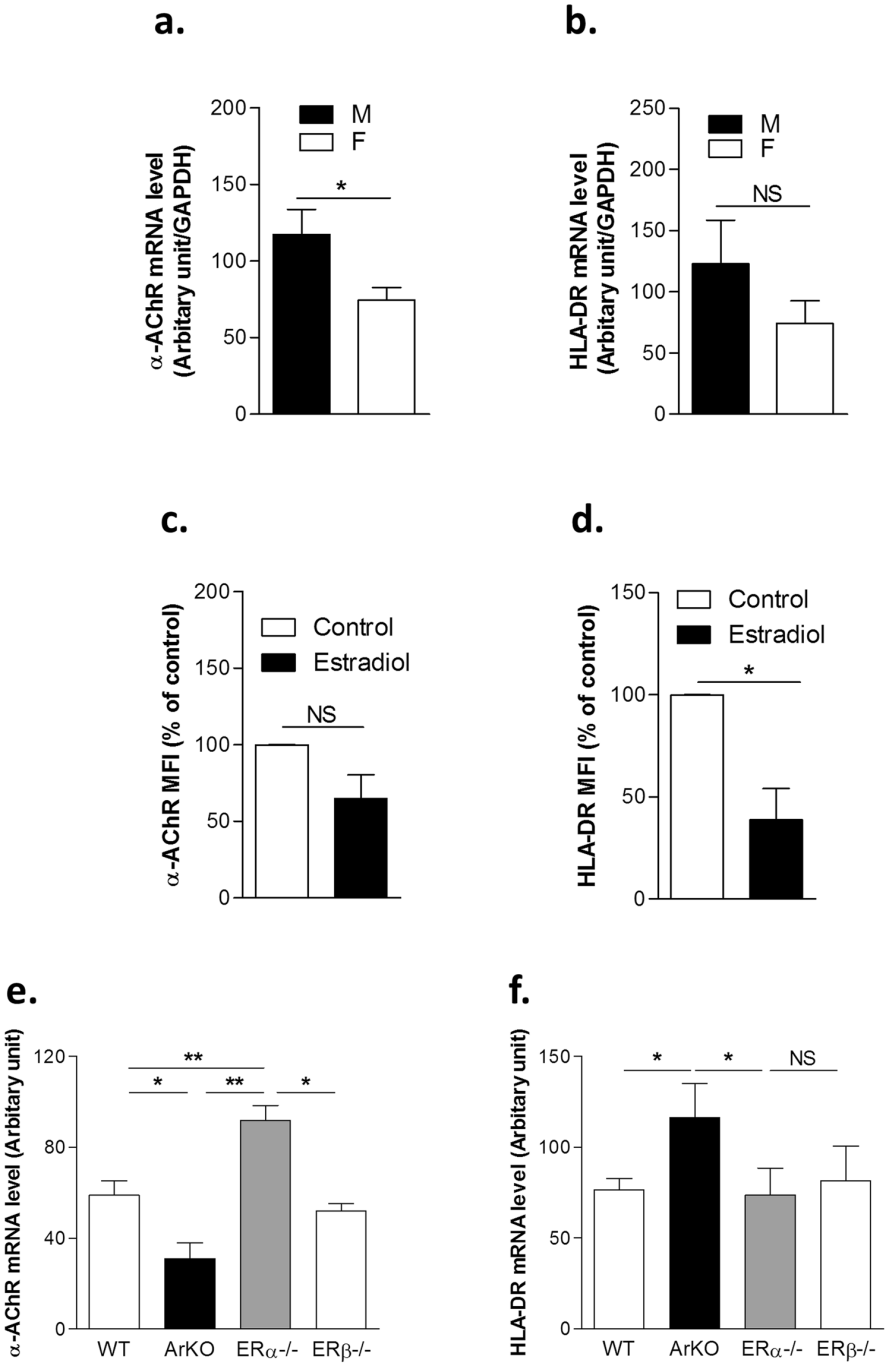
Modulation of α-AChR and HLA-DR expression in human and mouse thymuses and by β-estradiol in human primary cultured TECs. mRNA expression levels of α-AChR (**a**) and HLA-DR (**b**) in normal human male and female thymuses. Effect of 17-β estradiol (10^−8^M) on α-AChR (**c**) and HLA-DR (**d**) protein expression in human primary TECs. mRNA expression levels of α-AChR (**e**) and MHC II (**f**) in thymuses of C57Bl6J, Aromatase knock-out (ArKO), estrogen receptor α KO (ERα−/−) and estrogen receptor β KO (ERβ−/−) female mice. (n > 4 for human and n > 3 for mouse thymuses). P values were obtained using Mann-Whitney test for (**a**,**b**); P values were obtained using the Wilcoxon test for (**c**,**d**); P values were obtained using the Student t test for (**e**,**f**).

Analysis of whole normal human thymuses by real-time qPCR showed a gender differential effect with women expressing significantly less α-AChR but also slightly less HLA-DR compared to men (Fig. 1a,b). The effect of estrogens on the expression of these two molecules was analyzed in thymic epithelial cells that express AChR^26^ and MHC class II^27^, and are involved in thymic tolerance processes. Our data demonstrated that estrogens decreased the expression of α-AChR and HLA-DR proteins in cultured thymic epithelial cells (TECs) (Fig. 1c,d). This estradiol effect on the expression of α-AChR is significant at physiological doses (10^−8^ to 10^−9^) as observed in the dose effect curve (Supplemental Fig. S1). Altogether, these data suggest that estradiol may participate in the tolerization process for α-AChR subunit by regulating its expression and that of HLA-DR molecules.

To confirm the influence of estrogens on the expression of these molecules, we analyzed their mRNA levels in thymuses of mice deficient in estrogen receptor-α (ER-α) (principal isoform found in TECs^23^), estrogen receptor-β (ER-β) or aromatase (ArKO) (the enzyme involved in estrogen synthesis). As shown in Fig. 1e, the level of α-AChR was low in ArKO mice and significantly higher in ER-α KO mice compared to WT and ER-β mice, suggesting that estrogen effects were mediated through its nuclear receptor ER-α for α-AChR subunit expression in TECs. By contrast, MHC class II expression was not altered in ER-α, ER-β KO mice, but increased in ArKO mice (Fig. 1f), suggesting that estrogens may induce an ER-independent transduction pathway to control MHC II expression.

Altogether these data corroborate what we have already published about women after puberty, that display a decreased capacity in TECs to express tissue-specific antigens regulated by autoimmune regulator among them α-AChR^19^.

### Estrogen effects on chemokine expression in the thymus

**Figure 2:**
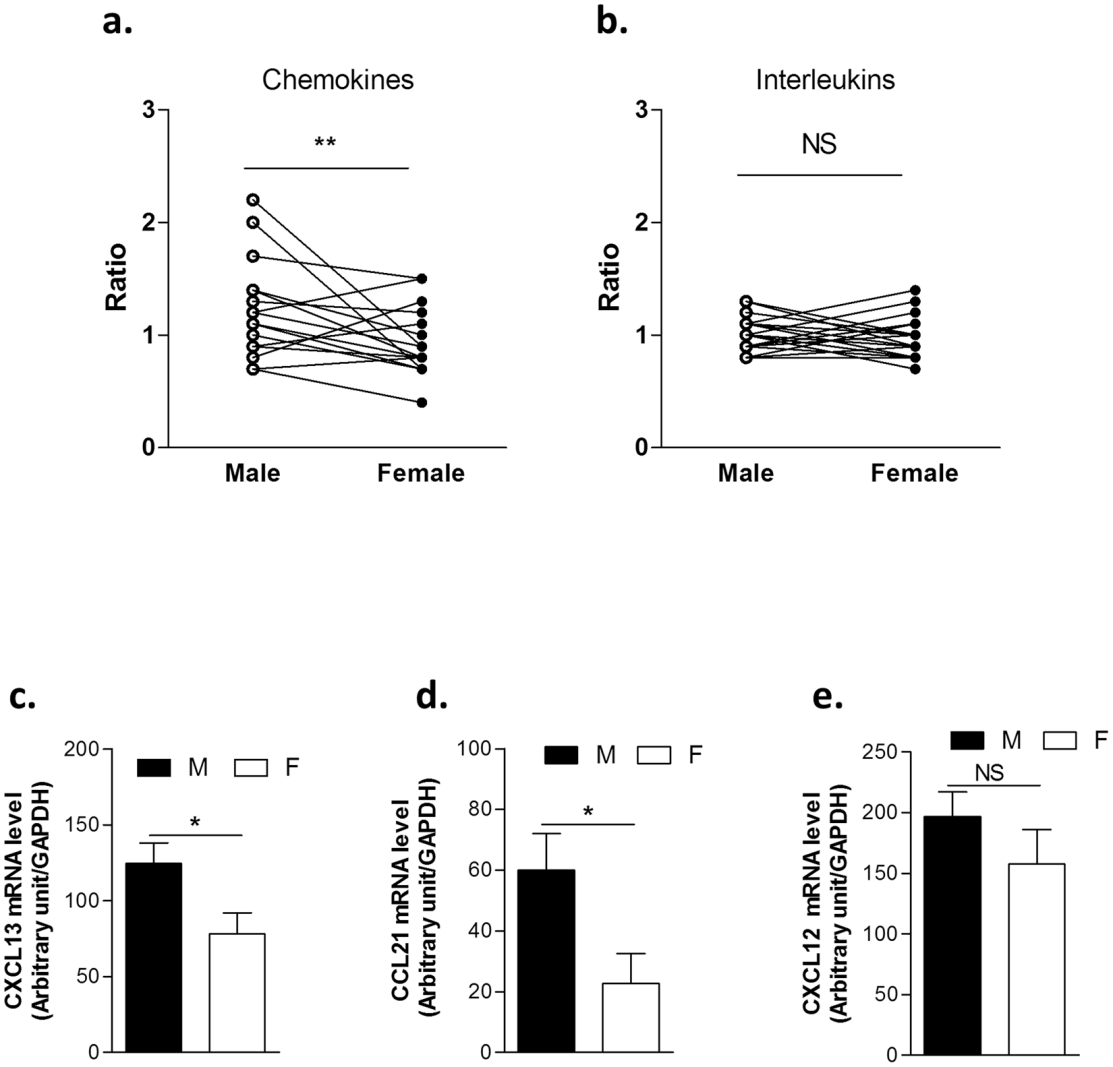
Analysis by microarray and RT-PCR of cytokine gene expression in male and female thymuses. Expression ratios of chemokine (**a**) and interleukin (**b**) genes spotted on the arrays for man and woman adults, compared to a thymic reference composed of thymuses from female babies. Each dot corresponds to the ratio of the median of five and four replicate arrays respectively for women, and men for a given gene. mRNA expressions of CXCL13 (**c**), CCL21 (**d**) and CXCL12 (**e**). n > 4 individual thymuses, aged from 17 to 44 years. P values were obtained using the Wilcoxon test for (**a**,**b**), and the Mann-Whitney test for (**c** to **e**).

We then wondered whether women exhibit differences in signaling molecules underlying the physiopathologic events occurring in the thymus of MG patients. We conducted a pan-genomic expression analysis using the “Human 1 cDNA arrays from Agilent” to compare thymic tissues from young men and women as detailed in Dragin *et al*.^19^. Analysis of the expression of all chemokines included in the arrays showed a significant difference between men and women (paired analysis, p < 0.01) (Fig. 2a) while no significant difference was observed for the interleukin family (Fig. 2b) (Supplemental Table S1a and b). Among the 17 chemokines included in the array, 14 were more expressed in males than females (Supplemental Table S1a). Moreover, a similar analysis on clusters of differentiation markers (Supplemental Fig. S2a) and keratins (Supplemental Fig. S2b) that illustrates the distribution of T-cell populations and epithelial cells, respectively, was performed. No differential expression between men and women for these gene families was observed, suggesting that the difference in chemokine thymic expression between men and women was not due to differences in thymic cell populations. Besides, to validate the gender differential expression observed in the microarray, we performed by real-time qPCR on, CXCL13, CCL21 and CXCL12, chemokines involved in MG pathological process. We observed that CXCL13 and CCL21 expressions were significantly decreased in women as observed with the microarray results, while CXCL12 did not display gender bias expression in whole human thymuses of adult women compared to men (Fig. 2c–e).

Since our data showed a decreased expression of chemokines in female thymus, we then asked whether estrogens could be responsible for these reduced chemokine expressions.

**Figure 3:**
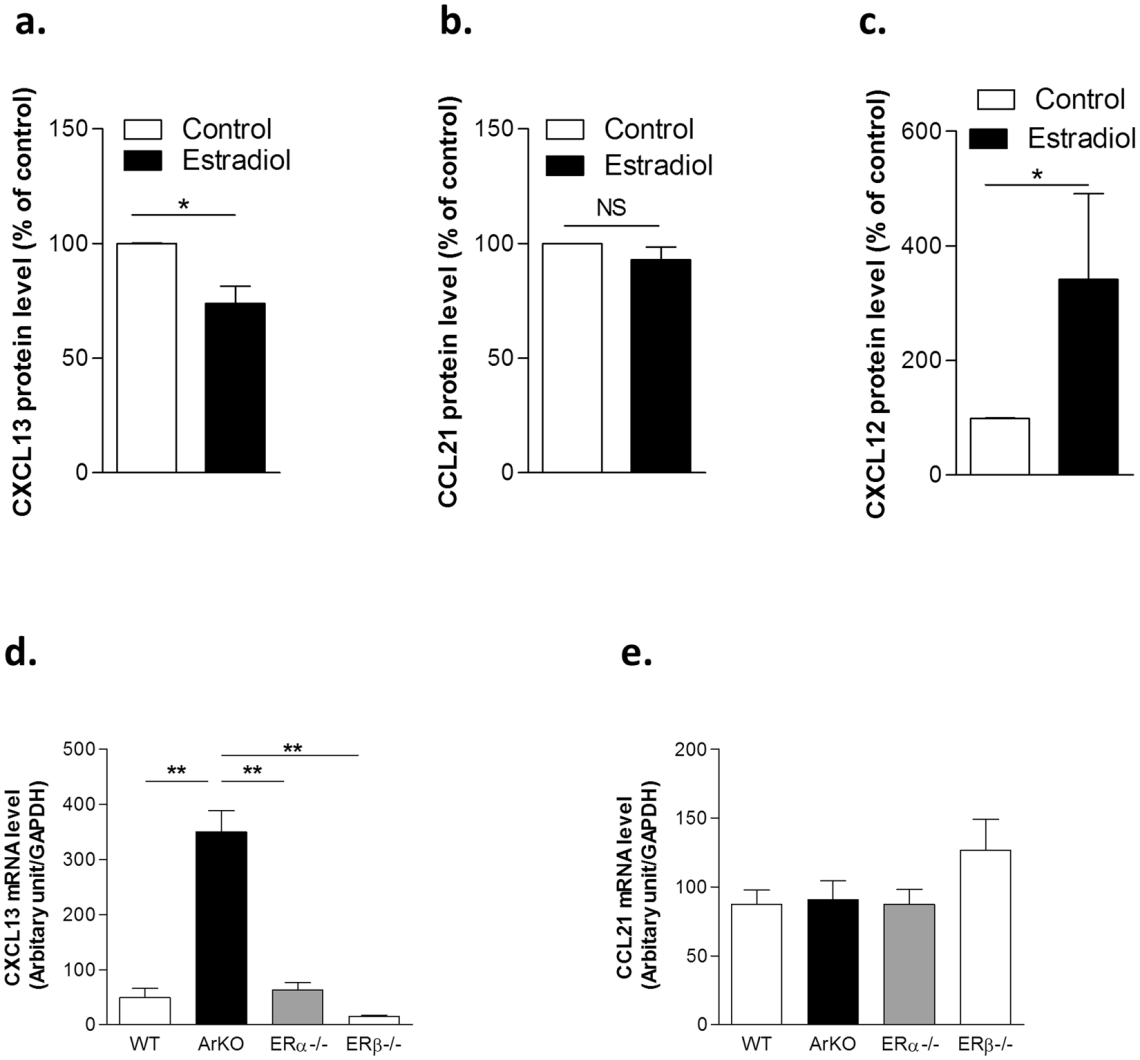
Modulation of chemokine expression in mouse thymuses and by β-estradiol in human primary cultured TECs. Human primary TECs were stimulated for 24 hours in the presence of 17-β estradiol (10^−8^M). Effects of estradiol on CXCL13 (**a**), CCL21 (**b**) and CXCL12 (**c**) protein expression by cultured primary human TECs. mRNA expression levels of CXCL13 (**d**) and CCL21 (**e**) in thymuses of C57Bl6J, aromatase knock-out (ArKO), estrogen receptor α KO (ERα−/−) and estrogen receptor β KO (ERβ−/−) female mice. Results are the mean values ± SEM. n = 5 different TEC supernatants for protein analysis and n > 3 different mouse thymuses per strain. P values were obtained using the Wilcoxon test for (**a** to **c**); P values were obtained using the Student t test for (**d**,**e**).

As shown in Fig. 3, in human primary TECs, a physiological dose of estradiol (E2) induced a significant decrease in protein expression of CXCL13 while no effect was found for CCL21 (Fig. 3a,b respectively), suggesting that lower level of CXCL13 in females compared to males could be due to the effects of estrogens (Fig. 3a). Of note, estradiol effect on the expression of chemokines is significant at physiological dose (10^−8^ to 10^−9^ M) as observed in the dose effect curves (Supplemental Fig. S3). Indeed, we demonstrated that normal physiological doses inhibited the expression of α-AChR, CXCL13, CCL21 while they upregulated CXCL12 expression in primary human TECs. Surprisingly, although no gender differential expression was found for CXCL12, estrogens up-regulated in TECs, its protein expression (Fig. 3c). Of note, these data corroborate previous findings displaying, in other tissues, upregulation of CXCL12 by physiological concentrations of estrogen through ER-α^28,29^.

In addition, the analyses of chemokine mRNA levels in the thymus of ER-α KO, ER-β KO or ArKO mice revealed that CXCL13 mRNA expression (Fig. 3d) was increased in ArKO mice but unchanged in ER-β KO mice. These data strongly suggest that CXCL13 transcript level was down-regulated by estrogens, independently to its ER-α nuclear receptor. By contrast, CCL21 expression was not altered in the mutated mice, suggesting that CCL21 expression is probably related neither to estrogens nor its nuclear receptors (Fig. 3e).

### Effect of inflammatory signals with estrogens on HLA-DR, α-AChR and chemokine expression

We have previously demonstrated a chronic inflammation in MG hyperplastic thymuses characterized by an overexpression of cytokines, in particular IFN γ and TNF α^30^. Since young female MG patients are prone to develop hyperplastic thymuses, we investigated here the interplay between estrogens and a pro-inflammatory environment. In order to mimic the inflammatory MG thymic condition that occurred in female.

**Figure 4:**
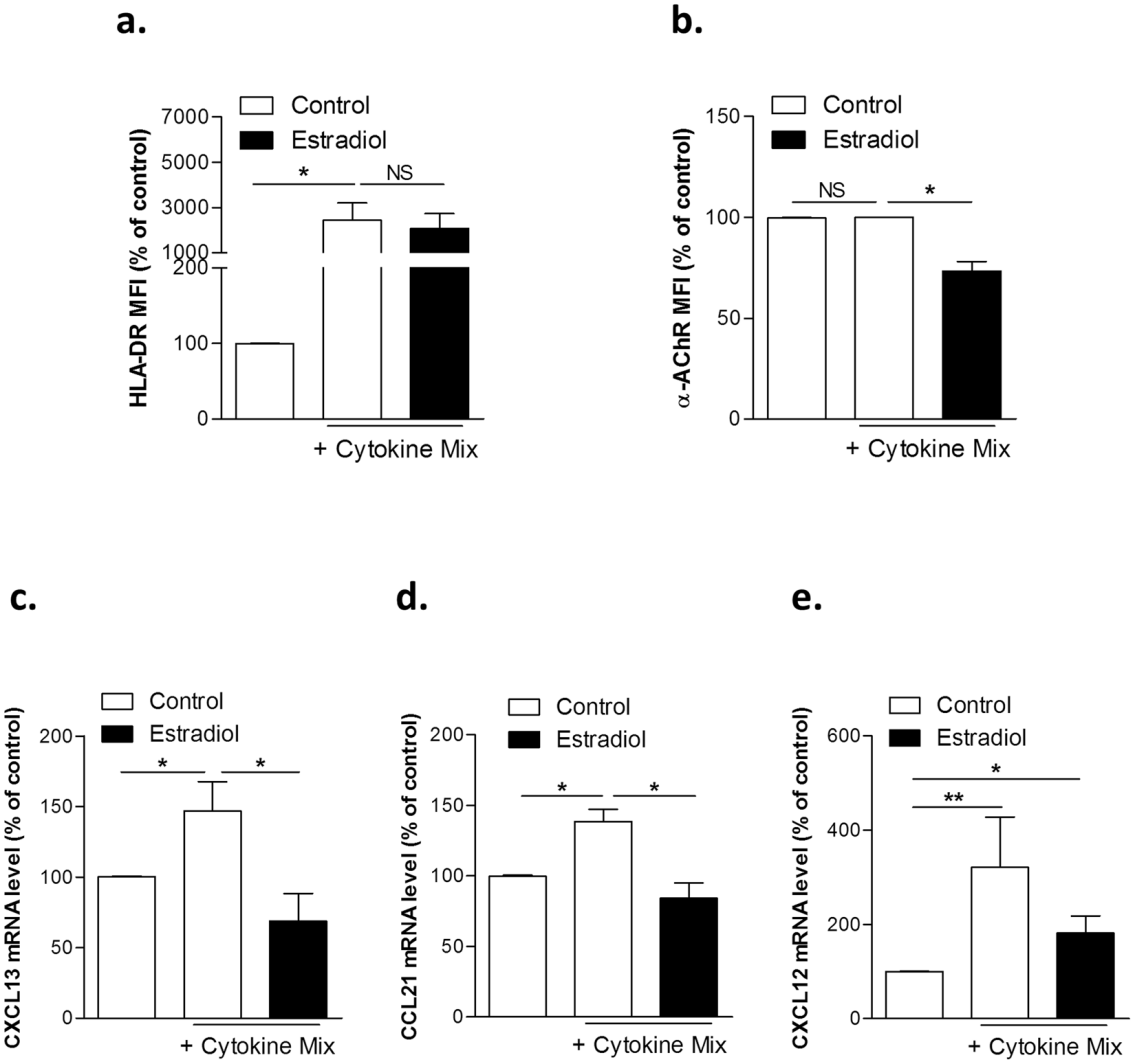
Modulation of HLA-DR, α-AChR, CXCL13, CCL21 and CXCL12 expression in human primary TECs by pro-inflammatory cytokine mix with β-estradiol. Effects of a cytokine mix with 17-β estradiol (10^−8^M) on HLA-DR (**a**), α-AChR (**b**), CXCL13 (**c**), CCL21 (**d**) and CXCL12 (**e**) expressions in human primary TECs. HLA-DR and α-AChR protein levels were analyzed by flow cytometry. Primary cultured TECs were obtained from at least five different donors. P values were obtained using the Wilcoxon test.

In primary cultured human TECs, the inflammatory mix that mimics MG thymic environment^31^ drove a significant expected and prominent upregulation of HLA-DR expression, an effect unchanged in the presence of estradiol (Fig. 4a). However, the cytokine mix combined with estradiol induced a reduced α-AChR expression (Fig. 4b).

The analysis of the chemokines at the mRNA level revealed that the cytokine mix caused an increased expression of CXCL13, CCL21, and CXCL12, and estrogens limited this increase (Fig. 4c–e). However, the changes at the protein level were less striking, except for CXCL13 that was significantly reduced in the presence of inflammatory cytokines (Supplemental Fig. S5). The apparent contradiction between the increased mRNA and decreased protein level could be due to a higher degradation or a reduced secretion of the proteins. Of note, the effect of single cytokine exposure was analyzed (Supplemental Fig. S4. We observed that IFNγ effect was the highest for CXCL13 and TNF-α and IL-1β were the highest for CXCL12. As previously observed, combined different cytokines lead to a stronger effect while cytokines are used in single. A synergistic or exacerbation is then observed on the cytokine expression.

**Figure 5:**
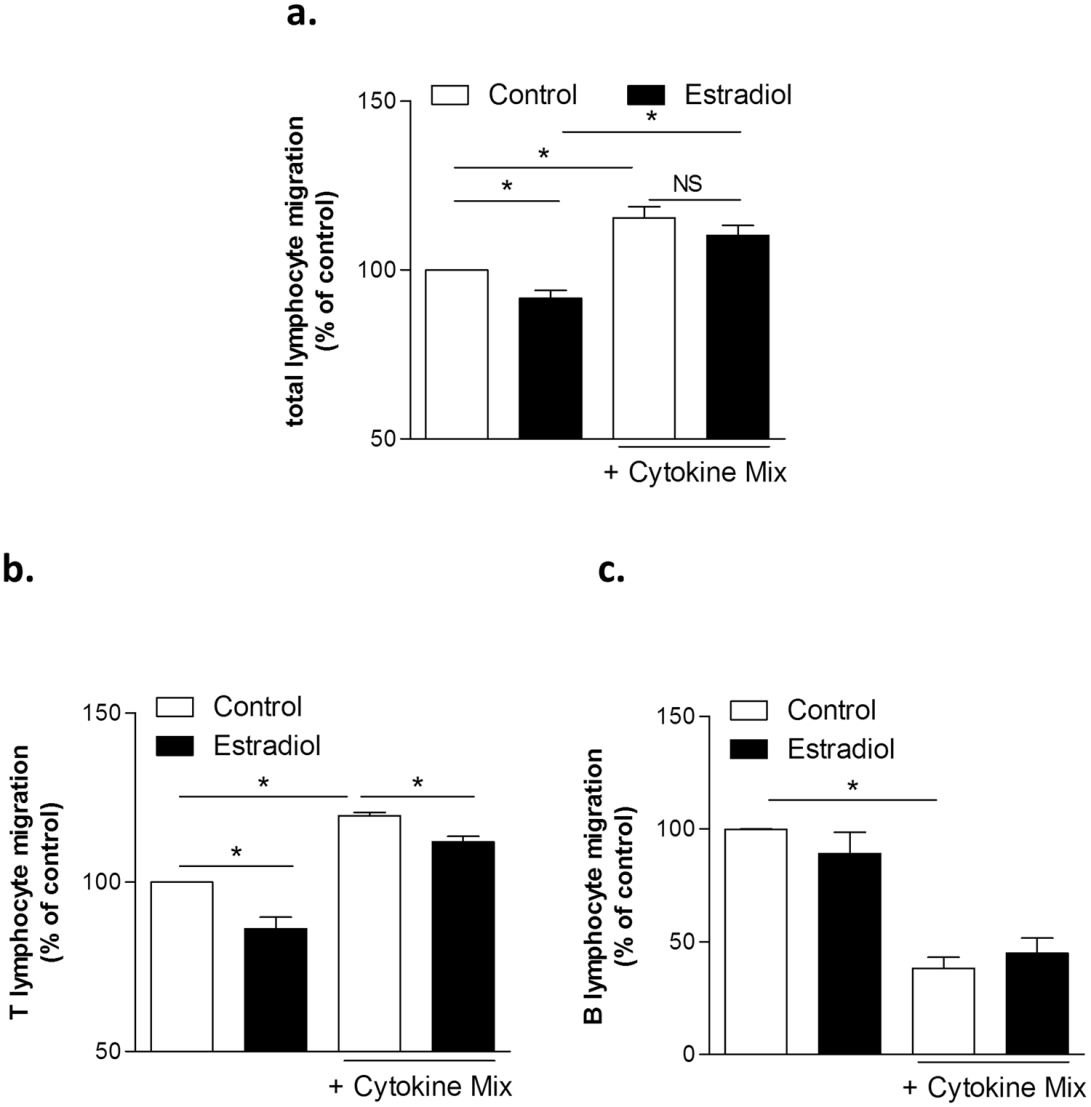
Analysis of Chemotactic properties of supernatants of human primary TECs treated with β-estradiol with or without a pro-inflammatory cytokine mix. Chemoattraction of PBL (**a**), T lymphocytes (**b**) and B lymphocytes (**c**) by supernatants of primary human TEC cultures treated with 17-β estradiol (10^−8^ M) with or without a mix of pro-inflammatory cytokines (IFN-γ, IL-1β, and TNF-α) for 24 hours. Results are expressed as the percentage of migrating cells through the well (±SEM). Each experiment was normalized to 100 for untreated cells. n = 4 to 6 different blood donors. P values were obtained using the Wilcoxon test.

We next wondered whether this modulation might impact or modify the chemoattractant capacity of human TECs. We found that supernatants from TECs treated with estrogens exhibited a significant decreased chemotactic activity on peripheral blood leukocytes (PBLs) (Fig. 5a), an effect likely due to diminished migration of T (Fig. 5b) and not of B lymphocytes (Fig. 5c). Altogether, these data demonstrated that estrogens could decrease chemokine expression by TECs and then as a consequence reducing TEC ability to recruit T cells. In the presence of the inflammatory mix, the inhibitory effect of estradiol on chemotactism was less pronounced on total PBLs but still significant on T lymphocytes (Fig. 5a–c).

Together, by controlling the expression of some MG-related molecules, even in inflammatory conditions, estrogens can limit the inflammation burst. Interestingly, the effect of estrogens was detected only when the inflammatory mix induced small expression changes.

### Estrogens upregulate the expression of Type I interferon and related molecules in human primary thymic epithelial cells

The results above did not provide an explanation for the higher frequency of follicular hyperplasia in females that is associated with higher expression of CXCL13 and CCL21. Among molecules able to upregulate the expression of these chemokines, we previously reported that Type I interferon is a potent regulator of CXCL13 and CCL21 in primary cultured epithelial cells and lymphatic endothelial cells, respectively^32^. Others authors have demonstrated that IFN-I production or expression is regulated by estradiol^33^ in natural killer cells^34^, lymphocytes^35^, and B cells^36^. Moreover, estradiol regulation of IFN-I signaling participates to the gender bias disease development as already demonstrated by Choubey et coll^37^. We thus asked whether estrogens could affect the expression of type I interferon in human primary TECs.

**Figure 6:**
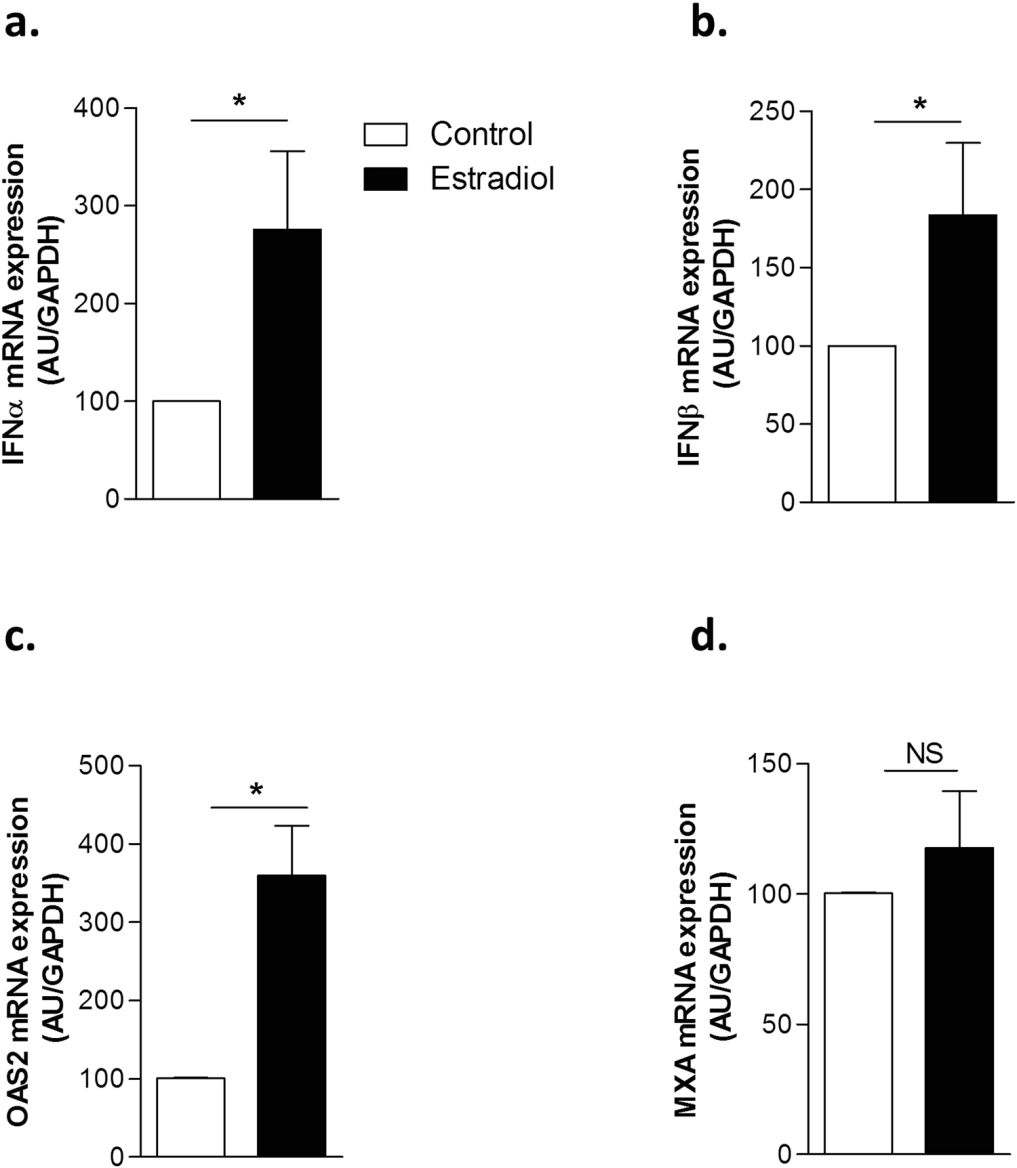
Modulation of interferon α, β, OAS2 and MXA gene expression in human primary cultured TECs by β-estradiol. Effects of 17-β estradiol (10^−8^M) on IFNα (**a**), IFNβ (**b**), OAS2 (**c**) and MXA (**d**) mRNA expression in human primary TECs. Total mRNA were extracted from four different cultured human TECs treated for 24 h. P values were obtained using the Mann-Whitney test.

We observed that estrogens stimulated expression of IFN-α and -β, (Fig. 6a,b respectively). Moreover, interferon-related genes such as OAS2 and MXA displayed also, but to a lesser extent, an estrogen modulation effect (Fig. 6c,d). These observations corroborate findings observed in other cell types showing that estrogens can stimulate the type I interferon pathway^38^. These data demonstrate the complexity of estrogen effect and its diverse roles depending on the environment.

## Discussion

Numerous studies have demonstrated that females are more susceptible to autoimmune diseases. Two groups have recently cleared up this evidence highlighting a guilty contribution to sex hormone by their involvement in the regulation of AIRE, a molecule with a pivotal role in the mechanisms of central tolerance^19,39^. In addition, another transcription factor, vestigial-like family member 3 or VGLL3, has recently been identified as a regulator of inflammatory network that promotes female-biased autoimmunity^40^. Women have also a stronger response to infection, inflammation or vaccination due to an increased antibody production^41^. Truffault *et al*. have recently demonstrated that 80% of thymic hyperplastic MG patients are females, suggesting that female related features such as hormonal status may be taking into account for the development of such disease phenotype^3^. Although the fact that women may be more sensitive than men to autoimmunity is understandable, it remains inexplicable why they preferentially develop thymic hyperplasia in MG. Therefore, the aim of this study was to investigate the role of estrogens in the pathogenic mechanisms associated with AChR sensitization and thymic hyperplasia, by studying the expression of MG-associated molecules including the biological events involved in GC development such as chemokine expressions.

### Estrogens and pathogenic mechanisms of MG

Estrogen display pro-inflammatory as well as anti-inflammatory effects through cytokine production regulation that depend on cell types, estrogen doses and the environment^42–46^.

Estrogens are potent driver of regulatory T cells (Treg) by promoting their proliferation^47^, by enhancing their suppressive activity^47^ through the activation of the programmed cell death protein 1 (PD1) pathway^48^ and by stimulating IL-10 secretion^49^. Our study confirmed the anti-inflammatory properties of estrogens and demonstrated that most of the molecules involved in the MG autoimmune response and the generation of GCs were down-regulated by estrogens. Interestingly, comparable estrogen effects have been shown in various human cell types among them human endometrial epithelial cells^50^, vaginal cells^51^ and intrahepatic biliary epithelial cells^52^ for which β-17 estradiol inhibited HLA-DR expression at transcript and protein levels. One of the main TEC functions is to express tissue-specific antigens^53^ such as α-AChR to set up the tolerance. This process of negative selection leads to the deletion of T-cells that strongly react with autoantigens presented by the medullary TECs through MHC class II^27^. We showed here that estrogens inhibited the expression of α-AChR and HLA-DR in TECs, suggesting that estrogens may alter the tolerization process, and favor the environment for an autoimmune response against α-AChR. Hence, these data suggest that estrogens in resting conditions create a “Yin/Yang” balance in the thymus by decreasing the expression of most chemokines that may reduce the autoimmune response but in same time alter the efficiency of the negative thymic selection.

Since estrogens display anti-inflammatory features^54^, one could have expected that estrogens down-regulate the expression of the genes involved in the anti-AChR autoimmune response in MG patients. The facts are in contradiction with this hypothesis since MG thymus displays inflammation and germinal centers especially in females^3^. One possible explanation for this discrepancy is related to the finding that estrogens can promote IFN-I expression in TECs. Since type I interferon can stimulate its own production in an autocrine manner, and upregulate the expression of chemokines, it could explain the GC development in the thymus of females. These data corroborate previous studies that have shown in another cell types, a positive regulation control of estrogens on interferon type 1 and type 2 genes^37,55^ and then on interferon-related gene levels. More, estrogens and IFN activate signaling pathways that work together to modulate estrogen- or IFN-sensitive genes^37^. Our study shows that expression of HLA-DR and α-AChR in human primary TECs was upregulated by inflammatory cytokines, confirming previous studies^31^, and similarly to what has been found in inflamed intestinal epithelial cells^56^. As recently suggested elevated expression in “inflamed” TECs of α-AChR favors, through an antigen cross-presentation by dendritic cells, the AChR autosensitization found in MG thymus^57^. Altogether, these observations tend to demonstrate that, in resting conditions, a low expression of α-AChR and HLA-DR by TECs induced by estrogens may result in a less efficient tolerance process that facilitates an increased women susceptibility to MG. However, once the pathology occurs, estrogens impact is restrained by the inflammatory molecules that control the immune response. Finally, the activation of IFN-I production by estrogens could interfere in the pathogenic processes.

### ERE on gene promoter of molecules implicated in MG pathogenesis

To corroborate the *in vivo* results, we analyzed CXCL13, CCL21, CXCL12, α-AChR and HLA-DR human gene promoter by using a predictive tool for the transcription factor promoter region binding site, The Champion ChIP Transcription Factor Search Portal (Sabioscience) (Supplemental Fig. S6).

Analysis of promoter region of HLA-DR and α-AChR revealed the presence of regulatory transcription factor binding sites related to estrogens but also to NF-κB signaling pathways, which are highly activated in inflammatory conditions. In this context, although estradiol had a high downregulatory effect on HLA-DR and α-AChR subunits, in the presence of pro-inflammatory cytokines, the effects of estrogens were limited, indicating that the pro-inflammatory cytokines dominate for the expression of these genes. Indeed, the down-regulatory effects of estrogens were more striking, when the upregulatory effects of inflammation were limited. In the case of HLA-DR, cytokines increased the expression by 25 fold, while estrogens reduced it by a factor of 2.5. As a result, estrogens had no significant effect in the presence of inflammatory cytokines. This estrogen effect appeared to be, for HLA-DR, independent from ER-α, which may corroborate a possible activation of a ligand-independent genomic activation pathway through c-Jun N-terminal Kinase pathway^58^ and or histone acetylation modifications^52^. By contrast, for α-AChR, estrogen regulation appeared to be clearly mediated through ER-α. The promoter region of CCL21, CXCL13 displayed ERE or XRE (for the Aryl Hydrocarbon Receptor), sites that required a recruitment of ER-α validating the involvement of estrogens in the control of the expression of these genes. The presence of ERE, in chemokine promoters, emphasizes the ability of estrogen alone to modulate cytokine and chemokine expressions. However, estradiol-induced changes in CCL21 expression were very limited (decrease factor of 1.4) in TECs even though the effects remained significant in the presence of cytokines (decrease factor of 1.6). Of note, in MG thymuses, it has been demonstrated that CCL21 increase is due to their production by lymphatic vessels corroborating the limited estrogens effect observed in TECs^59^. Altogether, our data demonstrated that the influence of estrogens was highly dependent upon the power of the inflammatory effect on a particular gene.

### Estrogens transduction pathways and AIDs

One can speculate that if estrogens display duals operating rules, animal models with estrogen deficiency should display variable resistance to experimental MG. So far, data are not conclusive for the role of estrogen in the disease course. Delpy and colleagues have shown that estrogen administration aggravates EAMG symptoms in mice^60^ while in rat^61^ no estrogen effect was observed on the severity of muscle weakness. Moreover, female castration has no effect on the rat susceptibility to EAMG^62^. Recently, it has been demonstrated that ER-α KO mice display a similar susceptibility to EAMG compared to WT mice, and a preserved humoral and cellular immune responses to AChR except for the TNF-α response^63^. However, the classical EAMG mouse model mimics the muscle disease but does not reflects the human thymic features. A model that develops the thymic hyperplasic MG feature would be more helpful to validate or corroborate estrogens roles in this pathology.

Nevertheless, by using deficient mouse models for estrogens transduction pathways, several studies brought clues in the relationship between estrogens, autoimmunity and GC formation. Indeed, ER-α knockout mice exhibit immune complex-type glomerulonephritis, destruction of tubular cells and severe infiltration of B lymphocytes in the kidney^64^ while ER-β deficient mice develop a bone marrow hyperplasia resembling myeloproliferative disease^65^. Even a defect in estrogen production, with aromatase KO mice, spontaneously leads to severe autoimmune exocrinopathy resembling Sjogren’s syndrome characterized by signs of autoimmunity with lymphoproliferative phenotypes in bone marrow and spleen^66^. In these models, disequilibrium in estrogen transduction pathway appears to contribute to the apparition of autoimmune symptoms such as cell infiltrations or GC formation.

We can then suggest a similar mechanism in MG. Combined with the increased pro-inflammatory activity in the thymus^67^, an abnormal decreased level of estrogens^68^ in young MG female patients could be associated with a high chemokine and cytokine production leading to migration of B and activated T cells towards the thymus, and an efficient antigenic presentation could, in turn, leads to the formation of GCs. This hypothesis also fits with our previous work showing the increased expression of ER-α on thymic cells^23^. Indeed, high levels of estrogens downregulate the expression of ERs, so one can speculate that a defect of estrogens could be associated with higher ER expression and the development of B-cell hyperplasia, leading to the GC formation in the highly activated thymus of MG patients. Alternatively, estrogens at normal or high levels could indirectly affect thymic inflammation via a higher production of type I interferon, which influences the expression of molecules involved in GC development.

### Conclusion

Our results highlight the subtle effect of estrogens. In resting conditions, estrogens have a dominant regulatory effect. However in an inflammatory milieu, the effects of estrogens were modulated: when cytokines produced a strong regulation, estrogens were not able to overcome it; however, in the absence or for low cytokine effect, estrogen did effectively affect the expression of molecules involved in autoimmune responses. Here, we demonstrated that equilibrium between estrogens and inflammatory cytokines could occur based on the strength of their respective effect. Therefore, this suggests that the estrogen-induced low chemokine expression in woman thymuses is overpassed when stimuli activate inflammatory pathways such as IFN-I related one. However, estrogens can modulate IFN-I production. Consequently estrogens operated a twist to contribute to hyperplastic thymic in MG by sustaining the inflammatory pathway (Supplemental Fig. S7).

## Materials and Methods

### Human samples

Human thymic fragments (50–100 mg) were obtained from immunologically normal male and female patients (babies aged two days to 1-year-old, and adults aged 15 to 27 years old) undergoing corrective cardiovascular surgery at Marie Lannelongue Chirurgical Center (Le Plessis-Robinson, France). All tissue samples were fast frozen in liquid nitrogen within 30 minutes of their excision from patients for mRNA analysis or put in sterile RPMI medium for culture experiments.

C57BL/6 mice were purchased from Janvier Laboratory (Saint Berthevin, France). Aromatase knockout mice (ArKo) were generated by the disruption of the Cytochrome P450 19A1 gene (Cyp 19) and were kindly provided by Evan R. Simpson^66^. They were backcrossed under C57BL6 background. Mice were 6 to 10 weeks old (n ≥ 6 per group). ERα−/− and ERβ−/− mice^69^ backcrossed under C57BL/6 background (more than ten generations) were from Dr. Habert’s mice colony (CEA, Fontenay-aux-Roses, France) under material transfer authorization #2010-036 of “Institut Clinique de la Souris”, (Strasbourg, France). Male and female mice were 6 to 10 weeks old.

### Primary cell cultures

Primary human thymic epithelial cell (TEC) cultures were established following the protocol previously described^70^. To avoid phenol hormone-like effects, RPMI phenol free was used. The culture medium was supplemented with 20% horse serum (Life Technologies, Invitrogen Corporation, Cergy-Pontoise, France), 0.2% Ultroser G (Life Technologies, Carlsbad, USA), two mmol/liter l-glutamine, 100 IU/ml penicillin, 100 μg/ml streptomycin, and 5 μg/ml fungizone. After 7–10 days, the cells were washed with phosphate-buffered saline (PBS) and collected by adding 0.075% trypsin (Life Technologies, Carlsbad, USA) and 0.16% EDTA for 10 min at 37 °C. Cells were then seeded and allowed to attach to the flask for 24 h before treatment in culture medium was supplemented with 5% horse serum.

Cells were treated for 24 hours with 17-β estradiol at 10^−8^ M (Sigma) in the presence or absence of a mix of cytokines (1 ng/mL recombinant human interleukin1β (IL-1β) (Sigma, Saint Quentin Fallavier, France), 10 ng/mL recombinant tumor necrosis factor-α (TNF-α), and 500 U/mL recombinant human interferon-γ (IFN-γ) (Genzyme, Cergy Saint Christophe, France) conditions previously described in Nancy *et al*.^23^.

### RNA extraction and reverse transcription

Thymuses were homogenized with the FastPrep FP120 instrument (Qbiogen, Illkirch, France). Total RNA was prepared from the thymus and TECs using the trizol RNA Isolation kit (Invitrogen, Cergy-Pontoise, France). The quality and concentration of RNA were analyzed with a NanoDrop ND-1000 spectrophotometer (LabTech, Palaiseau, France). RNA samples presenting a minimal ratio of 1.9 and 2 for respectively 260/280 and 260/230 were also controlled on a denaturing agarose gel. When the samples were degraded even partially, they were excluded. Total mRNA (1 μg) was reverse-transcribed using the SuperScript II RT kit (Invitrogen Cergy-Pontoise, France) according to the manufacturer’s instructions.

### Microarray experiments

Microarray experimentation procedure has previously been described by Le Panse *et al*.^9,19^. To minimize inter-individual variation, microarray experiments were performed with pools of RNA prepared with the equal amount of total RNA extracted from 4 thymuses of female donors aged 15 to 19 years old or from 3 thymuses of male donors aged 15 to 27 years old. The female and male RNA pools were co-hybridized respectively five and four times with a thymic reference composed of 10 thymuses of female babies aged one week to 1-year-old. All total RNAs were purified on Qiagen columns (Courtaboeuf, France) and their quality was assessed on an Agilent Bioanalyzer (Massy, France).

The experiments were performed with the “Human 1” cDNA arrays from Agilent (G4100A) according to the manufacturer’s instructions by using 20 µg of total RNA.

### Quantitative Real-Time PCR

Gene expression was evaluated by quantitative real-time PCR performed using the LightCycler apparatus (Roche Diagnostics, Meylan, France) as previously described by Dragin *et al*.^19^. The primers used are listed in Supplemental Table 2.

Each PCR was performed using the Fast-start DNA Master SYBR Green I kit (Roche Diagnostics, Meylan, France) according to the manufacturer’s instructions with the following conditions: initial denaturation at 95 °C for 10 min, then 40 cycles at 95 °C for 15 s, 60 °C for 14 s and 72 °C for 10 s, and a final fusion curve at 65 to 95 °C for 1 min.

Each cDNA sample was run at least in duplicate mRNAs were normalized to GAPDH. mRNA were expressed as arbitrary units and are the mean values (±SEM). For primary human TEC analysis, mRNA expression was normalized to 100 for untreated cells.

### ELISA

The levels of CXCL13, CCL21, and CXCL12 were analyzed in TEC supernatants. Plates were coated overnight at 4 °C with 2.5 μg/ml of mouse anti-human CXCL13 antibody (MAB801) or chicken anti-human CCL21 antibody (AF336). Cell supernatants (1/100 dilution) or standards were incubated for 90 minutes at room temperature, and subsequently, 0.25 μg/ml of biotinylated anti-human IgGs and streptavidin-horseradish peroxidase were added.

Recombinant human CCL21 (366-6 C/CF) and CXCL13 (801-CX-025) were used as standards. Tetramethylbenzidine was used for color development, and plates were read at 450 nm using MRX reader DYNEX (Thermo Lab systems, Cergy-Pontoise, France). All antibodies were purchased from R&D systems (Lille, France).

### Flow cytometry analyses

To analyze α-AChR subunit that is expressed intracellularly in TECs, cells were fixed and then permeabilized using IntraPrepTM Permeabilization reagents (Beckman-Coulter, Villepinte, France) according to the manufacturer’s instructions. The permeabilized cells were then labeled with an anti-AChR antibody (clone mAB 35; Sigma, Saint Louis, USA). The staining was detected by FITC coupled-anti rat immunoglobulins (Valbiotech, Paris, France).

HLA-DR was analyzed in non-permeabilized TECs with a mouse anti-human HLA-DR conjugated to FITC (clone B8.12.2, Immunotech Marseille, France). In all flow cytometry analyses, the results show the median (±SEM) fluorescence intensity (MFI). For each experiment, the MFI was standardized to 100 for untreated cells.

### Chemotaxis assays

TECs were treated for 24 h, and then supernatants were collected. Chemotaxis assay was performed in transwell plates (Costar/Dutcher, Issy-Les Moulineaux, France). TEC supernatants were placed in the lower wells while PBMCs were seeded in the upper wells. After five hours of incubation, cells were collected from both lower and upper wells and labeled with PE-coupled anti CD19 and FITC coupled anti-CD3-antibodies (DAKO Cytomation, Les Ulis, France). PBMCs were counted by flow cytometry assay calibrated using control microbead CaliBRITETM (BD Bioscience, Le Pont de Claix, France).

### Promoter sequence extraction and detection of ER-binding sites

The gene promoter sequences were obtained from the UCSC Genome Bioinformatics Site. We then used SABiosciences Text Mining Application, a tool for retrieving human/mouse putative orthologous promoter regions.

### Statistical analyses

Parametric or non-parametric test (Wilcoxon test for paired data, and Mann-Whitney test for unpaired data) were used to compare groups. Non-parametric tests were used thoroughly. However, because of the lack of power of these tests when the samples were too small (n < 4), in the few experiments with n < 4, the Student t test was used. The test is specified in the figure legend. Values are reported as Mean ± SEM. Statistical significance is recognized at p < 0.05. We used *GraphPad Prism 5* software to generate the graphs and to perform the statistical analyses. In all figures, the significance is displayed as stars, as follows: *P < 0.05; **P < 0.01; ***P < 0.001.

### Ethics approval and consent to participate

The use of human tissue included in the present study was approved by the local ethics committee (CPP, Kremlin-Bicêtre, France: agreement No. 06–018; CCP Ile de France Paris 6, France agreement No. C09-36).

All animals were handled under the Sonia Berrih-Aknin authorization from the French Ministry of Agriculture (agreement no. 075–1792) and according to the Animal Care and Use of Laboratory Animal guidelines of the French Ministry of Agriculture.

## Electronic supplementary material

supplemental
