# Author Correction: Balance between Estrogens and Proinflammatory Cytokines Regulates Chemokine Production Involved in Thymic Germinal Center Formation

**DOI:** 10.1038/s41598-018-23079-x

**Published:** 2018-05-22

**Authors:** Nadine Dragin, Patrice Nancy, José Villegas, Régine Roussin, Rozen Le Panse, Sonia Berrih-Aknin

**Affiliations:** 1 Inovarion, Paris, France; 2 Sorbonne Universités, UPMC Univ Paris 06, Paris, France; 3 INSERM, U974 Paris, France; 4 Department of Pathology, New York University, School of Medicine, New York, USA; 5 AIM, institute of myology, Paris, France; 6 Hôpital Marie Lannelongue, Le Plessis-Robinson, France

Correction to: *Scientific Reports* 10.1038/s41598-017-08631-5, published online 11 August 2017

In the original version of this Article, the author Rozen Le Panse was incorrectly indexed. Additionally, in the original version of this Article, Figure 6c was a duplication of Figure 6a. These errors have now been corrected in the HTML and PDF versions of the Article.

