# Supplementary material for: Balance between Estrogens and Proinflammatory Cytokines Regulates Chemokine Production Involved in Thymic Germinal Center Formation
Source: Sci Rep. 2017 Aug 11;7:7970. doi: 10.1038/s41598-017-08631-5 (PMC5554297; doi:10.1038/s41598-017-08631-5)
Supplement: Supplementary file 1 — supplemental [file 41598_2017_8631_MOESM1_ESM.pdf]

BALANCE BETWEEN ESTROGENS AND PROINFLAMMATORY CYTOKINES REGULATES CHEMOKINE PRODUCTION INVOLVED IN THYMIC GERMINAL CENTER FORMATION

Nadine Dragin[1, 2, 3,*], Patrice Nancy[4], José Villegas[2, 3, 5], Régine Roussin[6], Rozen Le Panse [2, 3, 5,] and Sonia Berrih-Aknin [2, 3, 5,]

[1] Inovarion, Paris, France

[2] Sorbonne Universités, UPMC Univ Paris 06, Paris, France

[3] INSERM U974, Paris, France

[4] Department of Pathology, New York University, School of Medicine, New York, USA

[5] AIM, institute of myology, Paris, France

[6] Hôpital Marie Lannelongue, Le Plessis-Robinson, France

[*] Corresponding author

Correspondence and Requests for materials should be addressed to:
Dr Nadine Dragin, Inovarion, Centre de Recherche en Myologie, UPMC / INSERM UMRS 974, Groupe Hospitalier Pitié-Salpêtrière, 105 Bd de l'hôpital, 75013 Paris, France, Tel: 00 33 (0)1 40 77 81 27, Fax: 00 33 (0)1 40 77 81 29; nadine.dragin-mamavi@upmc.fr

Keywords: HLA DR, acetylcholine receptor, CXCL13, Thymic epithelial cells, sex hormones, thymus

Supplementary material

Figure S1. Estradiol dose effects on α-AChR mRNA expression in human primary thymic epithelial cells (TECs).

Human primary TECs were treated for 24h with 10^{-10} to 10^{-7} M of 17-β estradiol. α-AChR mRNA level was quantified by real time PCR. Primary cultured TECs were obtained from different donors. The results are expressed as mean values (± SEM). P values were obtained using the Man Whitney test.; ** p<0.01

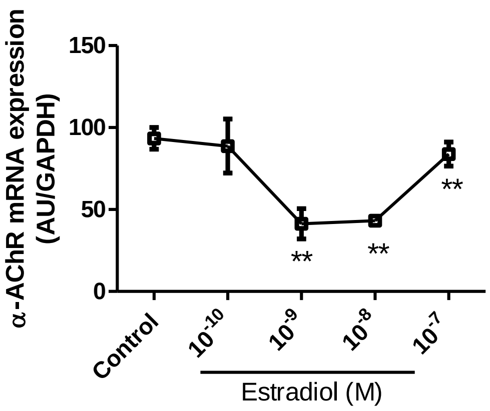

Figure S2. Compared expression of "clusters of differentiation" (CD) and keratins. mRNA expression ratios of cluster of differentiation (n= 71) (a) and Keratin (b) (n=26) genes spotted on the arrays for men and women in normal adult thymuses. The expressions were normalized and compared with a thymic reference composed of thymuses from female babies. Each dot corresponds to the median of ratios of five replicates for women, and four replicates for men for a given gene. P values were obtained using the Wilcoxon test.

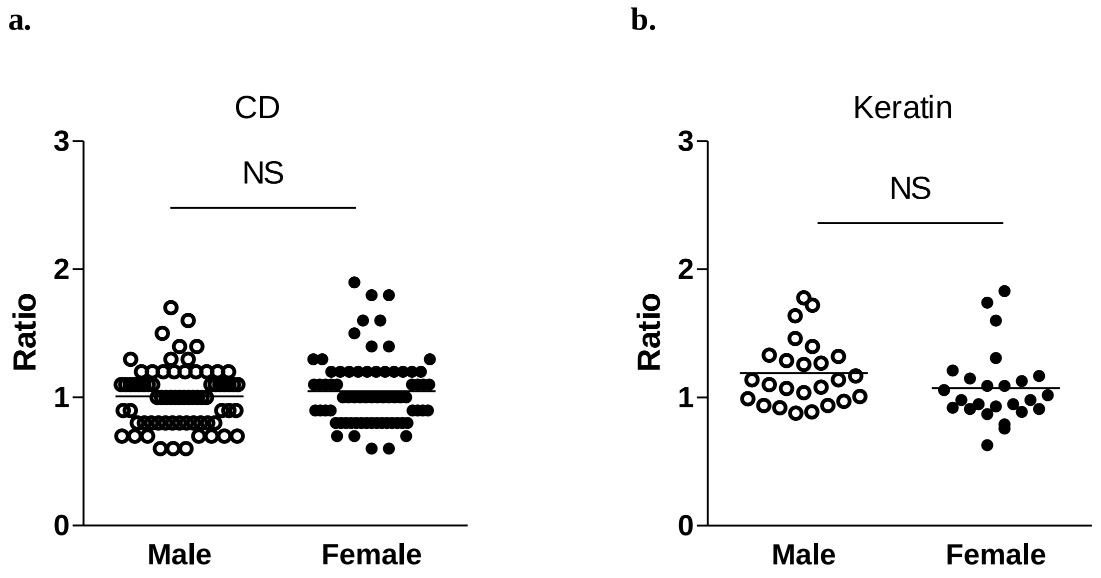

Figure S3. Estradiol dose effect on CXCL13, CCL21 and CXCL12 expression in primary human thymic epithelial cells.

Human primary TECs were treated for 24h with 10^{-10} to 10^{-8} M of 17-β estradiol. CXCL13 (a), CCL21 (b) mRNA levels were quantified by real time PCR. CXCL12 (c) protein levels were evaluated in the cell culture supernatants. Primary cultured TECs were obtained from different donors. The results are expressed as mean values (± SEM). P values were obtained using the Man Whitney test. ** $p<0.01$

a.

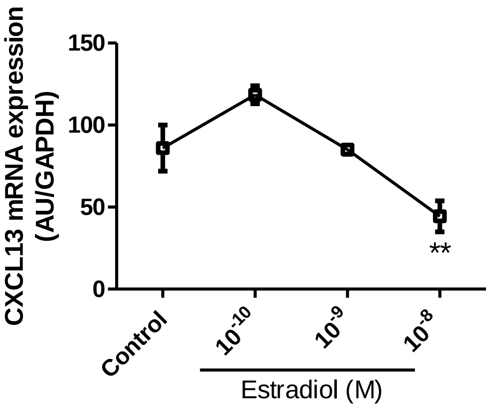

b.

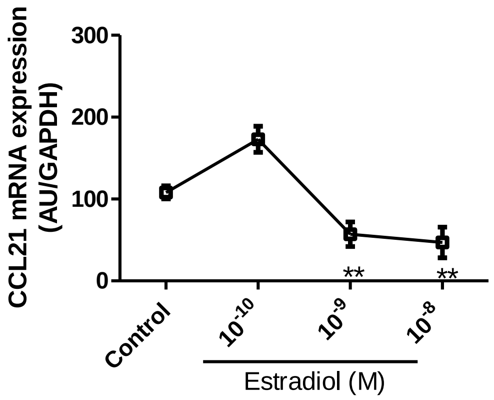

c.

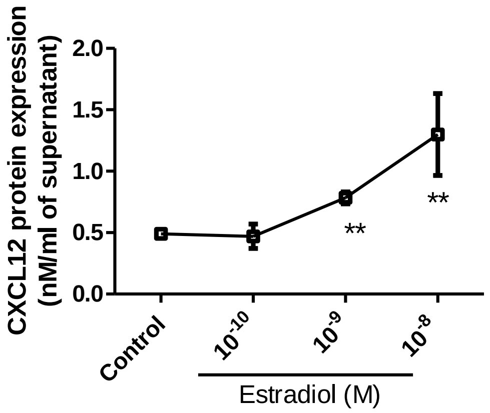

Figure S4. Cytokine single dose effect on CXCL13, CCL21 and CXCL12 expression in primary human thymic epithelial cells.

Effects of TNF-α (10 ng/mL), IL-1β (1 ng/mL recombinant) or IFN-γ (500 U/mL) at 24h of exposure on CXCL13 (a), CCL21 (b) and CXCL12 (c) protein levels in the supernatants of human primary TECs. Primary cultured TECs were obtained from different donors. The results are expressed as mean values (± SEM). P values were obtained using the Man Whitney test. * p<0.05; ** p<0.01

a.

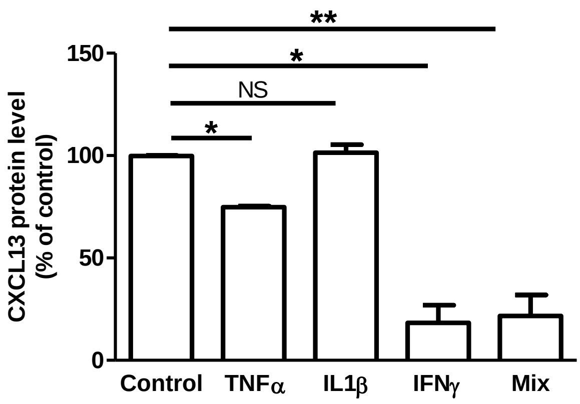

b.

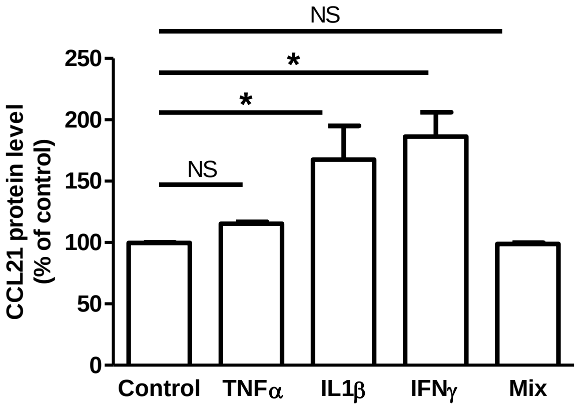

c.

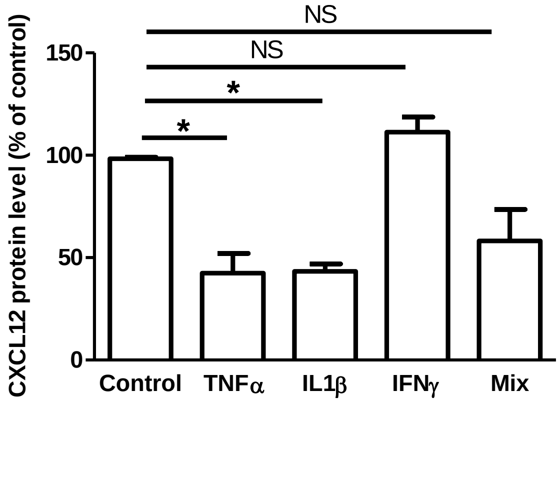

Figure S5. CXCL13, CCL21 and CXCL12 protein expression by human primary TECs treated with pro-inflammatory cytokines and with estradiol.

Effects of a cytokine mix (IFN-γ, IL-1β, and TNF-α) with 17-β estradiol (10^{-8}M) exposure on CXCL13 (a), CCL21 (b) and CXCL12 (c) protein levels in the supernatants of human primary TECs. Primary cultured TECs were obtained from at least five different donors. The results are expressed as mean values (± SEM). P values were obtained using the Wilcoxon test. * $p<0.05$; ** $p<0.01$

a. **b.** **c.**

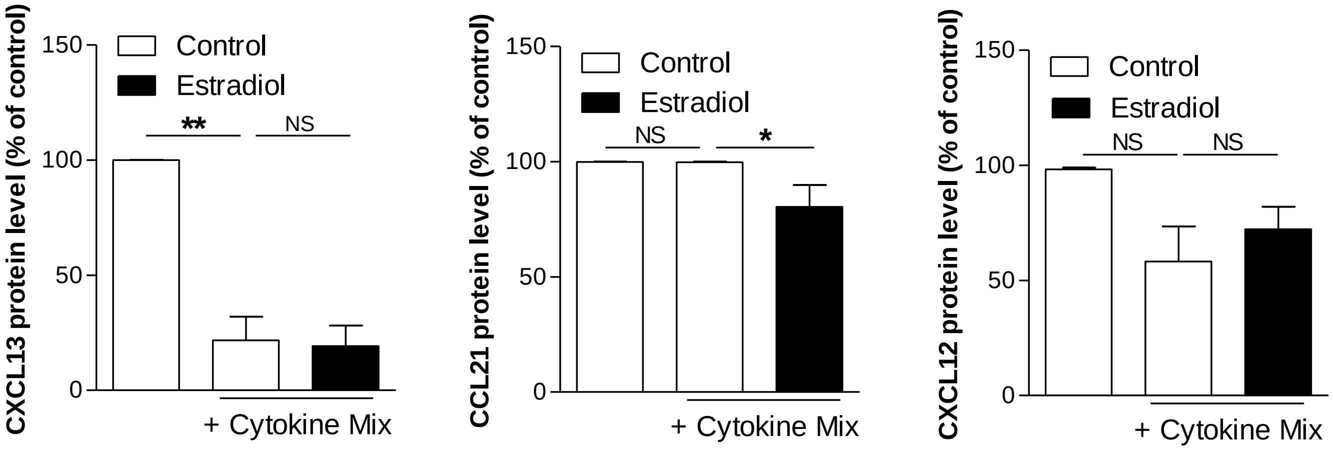

Figure S6.

Graphical representation of Transcription factor binding sites found in promoter of HLA DR, α-ACHR, CXCL13, CCL21 and CXCL12 genes. Graphic provided by The SABiosciences Champion ChiP Transcription Factor Search Portal that used the database known as DECODE.

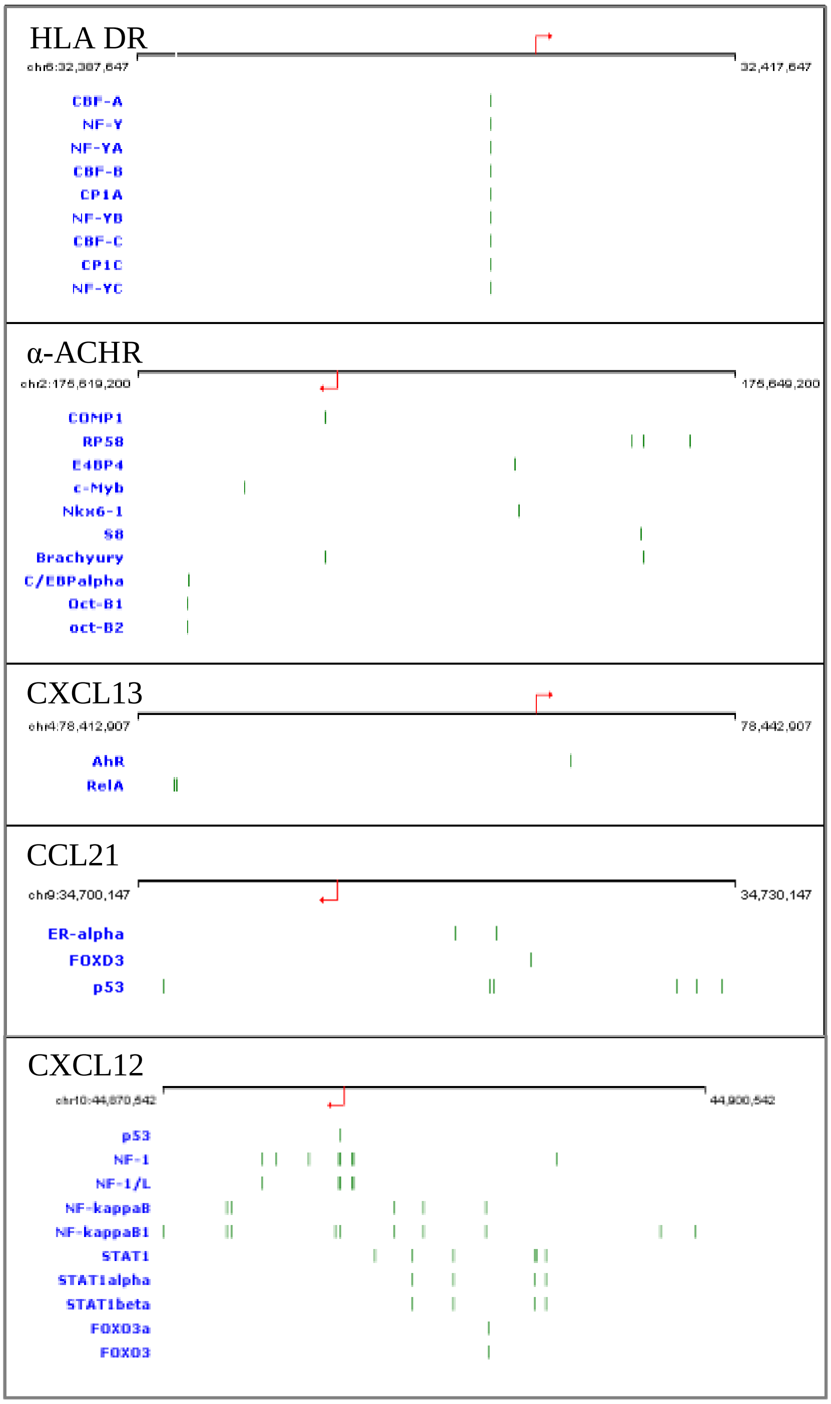

Figure S7. Summary of estrogen effects depending on steady or inflammatory environnement.

In steady state, estrogens (estradiol E2) contribute to a reduced tolerisation process to autoantigen such as α-AChR and to lesser chemokine expression. However, in a virus-induced inflammation milieu such as found in autoimmune $AChR^+$ MG thymuses, estrogen direct effects are overpassed by inflammatory pathways such as toll like receptor (TLR)/IFN and then estrogens collaborate with this pathway to stimulate expression of chemokines involved B cell chemoattraction and germinal center formation. Estrogens act as an "opportunist" guy that probes the environment and ajusts its effect to or with the combined stimulus.

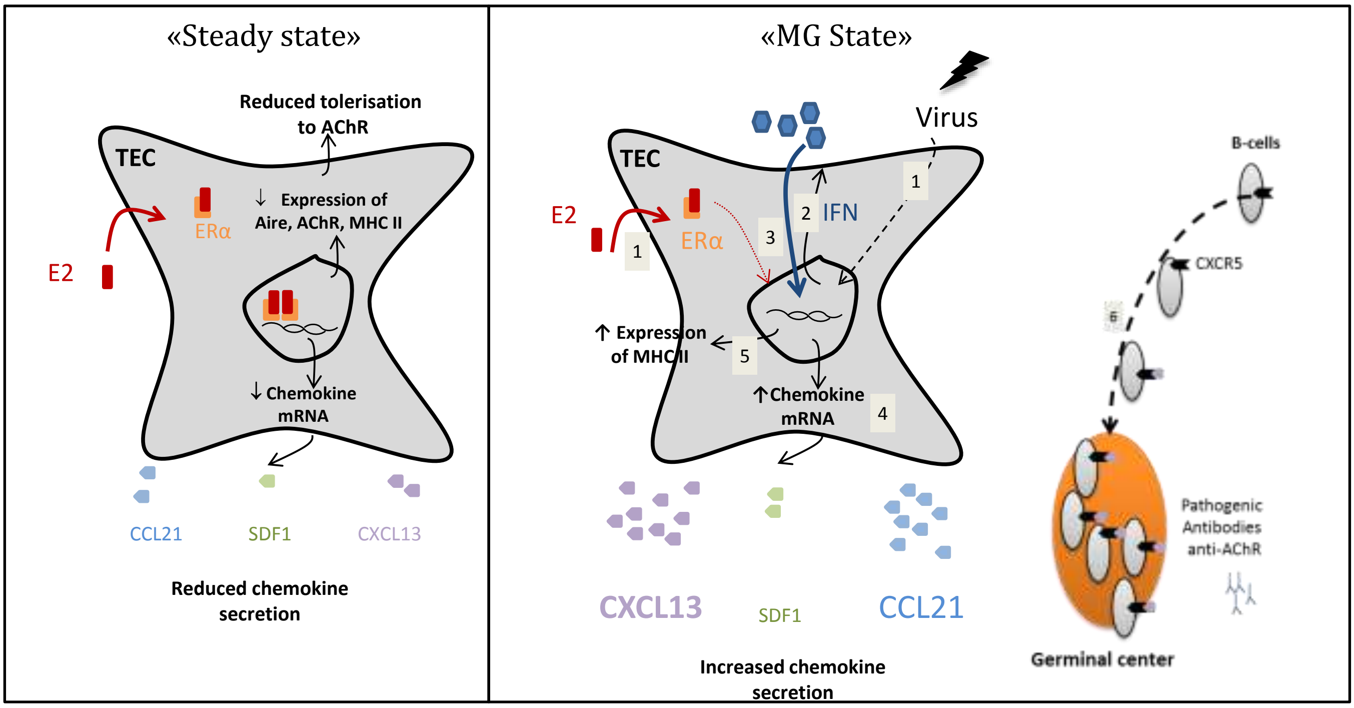

Table S1A: **list of Chemokine genes (n= 17) spotted on the arrays**

	Gene Name	Female	Male
1	CXCL3	0,7	**1,0**
2	CXCL6	0,7	**1,1**
3	CXCL10	**1,1**	0,9
	CXCL10	**1,3**	0,8
4	CXCL11	0,8	**1,1**
5	CXCL13	0,8	**1,1**
6	XCL2	0,7	**2,0**
7	CX3CL1	0,9	**1,2**
8	CCL4L2	0,9	**2,2**
9	CCL5	**1,5**	1,2
10	CCL7	0,8	**1,1**
11	CCL11	0,8	**0,9**
12	CCL15	1,2	**1,3**
13	CCL17	**0,8**	0,7
14	CCL18	0,4	**0,7**
15	CCL19	1,5	**1,7**
16	CCL21	1,0	**1,4**
17	CCL8	0,8	**1,4**

Table S1B: list of Interleukin genes (n= 21) spotted on the arrays

	Gene Name	Female	Male
1	Interleukin 1 alpha	0,7	**0,9**
2	Interleukin-1 homolog 1	0,8	**1,0**
3	Interleukin 1B	0,8	**1,1**
4	Interleukin 2	0,9	**1,1**
5	Interleukin 3	0,8	**0,9**
6	Interleukin 4	0,8	0,8
7	Interleukin 5	1,0	**1,2**
8	Interleukin 6 (complete cds)	**1,1**	0,8
	Interleukin6 (IFN beta 2)	1,0	**1,2**
9	Interleukin 7	**1,0**	0,9
10	Interleukin 8	**1,4**	1,1
11	Interleukin 11	1,0	**1,3**
12	Interleukin 12A	1,0	**1,1**
13	Interleukin 13	0,9	**1,0**
14	Interleukin 15	0,9	**1,3**
	Interleukin 15	0,7	**1,0**
15	Interleukin 16	**1,2**	0,9
16	Interleukin 18	**1,3**	1,0
17	Interleukin 20	**1,1**	0,8
18	Interleukin 24	**1,1**	0,9
19	Lymphotoxin beta	**0,9**	0,8
20	TNF-beta	0,8	**0,9**
21	GM-CSF	0,8	**1,0**

Table S2: list of primers used in the study

GENE NAME	SPECIES	PRIMER #1	PRIMER #2
28S	Human	GGTAGGGACAGTGGGAATCT	CGGGTAAACGGCGGGAGTAA
α-AChR		AAGCTACTGTGAGATCATCGTCAC	TGACGAAGTGGTAGGTGATGTCCA
CCL21		CAAGCTTAGGCTGCTCCATC	TCAGTCCTCTTGCAGCCTTT
CXCL13		CTCTGCTTCTCATGCTGCTG	TGAGGGTCCACACACACAAT
GAPDH		GCTGAGTACGTCGTGGAGTC	GATGATGTTCTGGAGAGCCC
IFNα		TCCTGCTTGAAGGACAGACA	TTTCAGCCTTTTGGAACTGG
IFNβ		ACGCCGCATTGACCATCTATG	CGGAGGTAACCTGTAAGTCTGT
HLA DR		TGGAGCAGATTAAACACGAGTG	CCGCCCGGAACTTTCTGAC
MXA		ACCTACAGCTGGCTCCTGAA	CGGCTAACGGATAAGCAGAG
OAS2		ACAGTCCTGCAGCGAAACTT	AGTGTCAAAATCCGGCACTC
CXCL12		TCAGCCTGAGCTACAGATGC	CTTTAGCTTCGGGTCAATGC
α-AChR	Mouse	GTGCTGGGCTCTTTCATCTC	TTCTGTGCGCGTTCTCATAC
CCL21		CCCTGGACCCAAGGCAGT	AGGCTTAGAGTGCTTCCGGG
CXCL13		TGAGGCTCAGCACAGCAA	ATGGGCTTCCAGAATACCG
GAPDH		ATCACCATCTTCCAGGAGCG	CCTGCTTCACCACCTTCTTG
HLA DR		CCACTGGACATGGAAGACCT	GACTTCATTTGCCGTGTCCT
CXCL12		GCTCTGCATCAGTGACGGTA	ATTTCGGGTCAATGCACACT
